# The Role of the Bone Marrow Microenvironment in the Response to Infection

**DOI:** 10.3389/fimmu.2020.585402

**Published:** 2020-11-25

**Authors:** Courtney B. Johnson, Jizhou Zhang, Daniel Lucas

**Affiliations:** ^1^ Division of Experimental Hematology and Cancer Biology, Cincinnati Children’s Medical Center, Cincinnati, OH, United States; ^2^ Department of Pediatrics, University of Cincinnati College of Medicine, Cincinnati, OH, United States

**Keywords:** bone marrow, niche, hematopoiesis, infection, microenvironment

## Abstract

Hematopoiesis in the bone marrow (BM) is the primary source of immune cells. Hematopoiesis is regulated by a diverse cellular microenvironment that supports stepwise differentiation of multipotent stem cells and progenitors into mature blood cells. Blood cell production is not static and the bone marrow has evolved to sense and respond to infection by rapidly generating immune cells that are quickly released into the circulation to replenish those that are consumed in the periphery. Unfortunately, infection also has deleterious effects injuring hematopoietic stem cells (HSC), inefficient hematopoiesis, and remodeling and destruction of the microenvironment. Despite its central role in immunity, the role of the microenvironment in the response to infection has not been systematically investigated. Here we summarize the key experimental evidence demonstrating a critical role of the bone marrow microenvironment in orchestrating the bone marrow response to infection and discuss areas of future research.

## Introduction

The bone marrow microenvironment is the collection of cells and structures that together support blood cell production in the bone marrow. The architecture, composition, and function of the microenvironment has been recently reviewed in great detail (1, 2). The bone marrow architecture is defined by the enclosing bone tissue and the blood vessels that irrigate it. Small arterioles penetrate the BM through the bone and give rise to a dense network of sinusoids that drains through a central vein (3). The endothelial cells that form the BM vessels are major components of the microenvironment *via* their production of cytokines that support and regulate hematopoietic stem cells and other progenitors (4–6). Additional major sources of hematopoietic supportive cytokines are Ng2^+^ cells that ensheath arterioles (7–9); a network of perivascular cells [defined as LepR^+^, Cxcl12-abundant reticular cells, or Nestin-GFP^dim^ cells depending on the genetic reporter used to prospectively isolate them (3–6, 10–13)]; and non-myelinating Schwann cells (14). Stepwise hematopoiesis takes place in the space between these stromal cell types. Additionally, many hematopoietic and non-hematopoietic (stromal) cells cooperate to regulate blood cell production. Examples of these include: megakaryocytes that—in addition to platelet production—function by restricting hematopoietic stem cells (HSC) proliferation (15–17); macrophages which provide a niche for red blood cell production [reviewed in (18)] but also crosstalk with stromal (non-hematopoietic) cells (19–21) and neutrophils (22) to regulate HSC release into the circulation; and dendritic cells which function as antigen-presenting cells, but also control HSC trafficking by targeting the endothelium (23). As the main source of immune cells, the bone marrow is a major player in -and target of- the response to infection [reviewed in (24–26)]. Here, we focus on the role of the microenvironment in orchestrating the response of the bone marrow to infection.

## Functional Consequences of Infection in the Bone Marrow

Below we summarize the diverse effects of infection on hematopoiesis and discuss the experimental evidence demonstrating the role of the microenvironment in orchestrating these responses. Note that these are not independent phenomena and, in many cases, take place simultaneously and are mediated by the same mechanisms. However, the BM response to infection is also pathogen-specific (27–29) probably due to differences in the signaling pathways used to detect the infection and pathogen dosage (27). The main BM responses to infection are:

1. Emergency myelopoiesis: Neutrophils, monocytes, and dendritic cells (DC) are consumed in great quantities during infection. Emergency myelopoiesis is the main mechanism used by the bone marrow to produce large numbers of myeloid cells to replenish those consumed in the periphery. Emergency myelopoiesis has been traditionally divided into **emergency granulopoiesis**—the process of emergency neutrophil production—and into **emergency mono/DC- poiesis**—the process of emergency production of monocytes and dendritic cells. It is characterized by proliferation and preferential commitment of multipotent progenitors toward myeloid fates and of lineage-committed progenitors toward neutrophil (emergency granulopoiesis) or mono/DC fates (emergency monopoiesis). Both are also associated with rapid release of hematopoietic progenitors and myeloid cells into the circulation (see point two below). Several studies support a specific role of the microenvironment in orchestrating emergency myelopoiesis. Lipopolysaccharide (LPS) triggers emergency granulopoiesis *via* toll-like receptor 4 (TLR4) activation. Boettcher et al., used bone marrow transplantation to generate chimeric mice in which TLR4—or its downstream adaptor Myd88—was knocked out in the stroma or in hematopoietic cells. These experiments showed that TLR4 expression in the stroma was necessary and sufficient to trigger emergency granulopoiesis in response to LPS. *Tie2-cre:Myd88^fl/fl^* mice, which lack MyD88 in hematopoietic and endothelial cells, are unable to induce emergency granulopoiesis after LPS treatment or *Escherichia coli* infection indicating that TLR4 signaling in endothelial cells orchestrates emergency granulopoiesis. In this setting, emergency granulopoiesis is likely mediated by endothelial cell secretion of granulocyte colony-stimulating factor (G-CSF), the major cytokine that supports granulopoiesis (30, 31). Similarly, the Kelsoe group showed that in response to the adjuvant alum, the BM initiated IL1 mediated emergency granulopoiesis (32). *Il1r1^−/^* ^−^ chimeric mice studies indicated that IL1 acted on stromal cells to trigger emergency granulopoiesis and G-CSF release (the specific identity of these G-CSF producing stromal cells is not known). Alum-induced emergency granulopoiesis was abrogated in G-CSFR knockout mice or in WT mice injected with neutralizing antibodies against G-CSF (32, 33). Interestingly- and in the same manuscript- the Kelsoe group showed that loss of neutrophils in the absence of inflammation was sufficient to induce G-CSF-dependent and -independent progenitor proliferation, similar to that observed during emergency granulopoiesis (33). This suggests that any infection that results in neutrophil depletion in the BM will also lead to G-CSF secretion by stromal cells which in turn will trigger emergency granulopoiesis. A critical role for neutrophils in mediating emergency granulopoiesis is further supported by the work of Kwak et al., who showed that, during inflammation induced by intraperitoneal injection of heat inactivated *E. coli-* myeloid cells (likely neutrophils), produced reactive oxygen species that stimulated emergency granulopoiesis (34).
  Emergency myelopoiesis can also be triggered—in a G-CSF independent manner—by stromal-mediated cytokine release. Chou et al., found that *Toxoplasma gondii* infection induced emergency granulopoiesis by expanding granulocyte monocyte progenitors and inhibited erythropoiesis by reducing megakaryocyte erythrocyte progenitors. *T. gondii-*induced emergency granulopoiesis was blocked in IL6 knockout mice (28). Chimeric mice studies showed that IL6 expression in stromal cells was necessary for emergency granulopoiesis. VCAM^+^PDGFRα^+^ mesenchymal cells—which are a subset of the LepR^+^ perivascular cells based on scRNAseq studies (35)—purified from infected mice expressed higher IL6 than those purified from control mice. These studies thus suggest that *T. gondii* induces IL6 release from perivascular cells which in turn re-programs hematopoietic progenitors toward neutrophil fates and emergency granulopoiesis (28). Schürch et al., found complex crosstalk between cytotoxic T- cells, mesenchymal stromal cells, and myeloid progenitors that drives emergency granulopoiesis in response to lymphocytic choriomeningitis virus (LCMV) infection (36). They found that transfer of LCMV-specific effector cytotoxic T cells caused expansion and proliferation of multipotent progenitors and myeloid progenitors and monocyte release into the circulation. Interferon gamma (*Ifng*) knockout T cells were unable to induce emergency myelopoiesis whereas *Ifngr^−/−^*mouse chimera studies showed that IFNGR expression in the stroma was required for emergency myelopoiesis. Only CD45^−^CD31^−^CD51^+^Sca-1^+^ stromal cells expressed functional IFNGR and these cells released IL6 upon IFNγ stimulation. Mouse chimeras lacking IL6 in stromal cells did not induce emergency myelopoiesis after cytotoxic T cell transfer (36). Together these studies indicate complex crosstalk where LCMV infection leads to the generation of LCMV cytotoxic T cells. These in turn produce IFNγ which targets bone marrow stromal cells to elicit IL6 production that then acts on hematopoietic progenitors to induce emergency myeloid production (36).
2. Mobilization: In addition to increased myeloid cell production, emergency myelopoiesis also encompasses the release of mature myeloid and hematopoietic stem cells and progenitors (HSPC) into the circulation. Mobilized progenitors can migrate to the spleen and other organs where they can differentiate *in situ*; this is advantageous in clearing local and systemic infections (27, 37). Many infections also cause stem and progenitor proliferation and loss of HSC (24–26). When HSC proliferate, they lose stem cell potential and can quickly become exhausted (38, 39). Infection-induced HSPC mobilization might also be used to replenish empty niches in distal bones thus maintaining the normal HSC pool (40). There is clear evidence indicating that hematopoietic cell mobilization in response to infection is regulated in a non-cell autonomous manner by the microenvironment.
  The chemokine CXCL12 and its receptor CXCR4 are the major signals regulating neutrophil (41, 42) and HSPC (43) retention in the bone marrow. CXCL12 is produced by perivascular stromal cells and endothelial cells (4, 6, 13, 43). G-CSF inhibits CXCL12 production in the BM and infections that cause increases in G-CSF, or reductions in CXCL12 (27, 30, 31, 44), will also elicit neutrophil and HSPC mobilization. Mechanistically, G-CSF functions by binding G-CSFR in a monocyte lineage hematopoietic cell. Through an unknown mechanism, these hematopoietic cells induce the downregulation of CXCL12 in BM stromal cells, triggering neutrophil and progenitor release to the circulation (21). A great example of this regulation is the work by Burberry et al. The bacterial wall contains LPS-sensed *via* the receptor TLR4 and the downstream mediators MyD88 and TRIF- and peptidoglycan-sensed *via* the receptors NOD1 and NOD2 and the adaptor RIPK2. Burberry et al., found that systemic infection elicited HSPC mobilization to the spleen. This mobilization was mostly abolished in *Trif^−/−^* and *Ripk2^−/−^* mice. Using bone marrow transplantation to generate chimeric mice with hematopoietic or stromal deletions of TLR4 and NOD1, they demonstrated that expression of these receptors in stromal cells was both necessary and sufficient for HSPC mobilization. Both receptors synergized to drive G-CSF expression by stromal cells (likely endothelial cells). Blocking G-CSF *via* antibody injection or by using G-CSFR^−/−^ mice completely abolished HSPC mobilization after infection (27). In an elegant experiment, the same group formally demonstrated that the mobilized HSPCs generate immune cells that function in clearing the infection. They transferred splenocytes from control or mobilized mice into recipients that were then infected with *E. coli*. The mice transferred with the mobilized splenocytes had dramatically lower levels of *E. coli* colonies in the spleen and liver (27).
  Mobilization of inflammatory monocytes in response to *Listeria monocytogene*s infection is also controlled by the stroma in a G-CSF-independent manner. Using *Ccr2^−/−^* and *Ccl2^−/−^* mice (*Ccl2 *encodes MCP1) Shi et al., found that low doses of LPS induced rapid monocyte mobilization in a CCR2/MCP1 dependent manner. Mouse chimeras showed that *Ccl2* deletion in non-hematopoietic cells completely abolished LPS-induced monocyte mobilization. Using an MCP1 reporter mouse, Shi et al., demonstrated that LPS quickly (2 hours) induces MCP1 expression in perivascular stromal cells that are tightly associated with sinusoids. Conditional *Ccl2* deletion in perivascular cells using *Nestin-cre* mice greatly reduced monocyte egress from the BM and reduced bacteria clearance in a model of *L. monocytogenes* infection (45).
  During homeostasis, several types of hematopoietic cells regulate HSPC release into the circulation. Bone marrow macrophages and monocyte-lineage cells specifically crosstalk with bone marrow perivascular cells promoting CXCL12 production. Loss of bone marrow macrophages leads to reductions in CXCL12 and HSPC release (19–21). In addition, trafficking and phagocytosis-by bone marrow and intestinal macrophages- also control HSPC release during homeostasis (22, 46). A recent study also showed that bone marrow dendritic cells regulate HSC release through the BM, likely by modulating permeability of sinusoids *via* CXCL1-CXCR2 signaling (23). Since infection causes massive changes in the numbers of these hematopoietic components of the microenvironment, it is highly likely that the same pathways will also participate in regulating HSPC mobilization during infection.
3. Hematopoietic injury: Infection frequently causes bone marrow aplasia and inefficient hematopoiesis as well as loss of functional HSC (24–26). While some of these are mediated by direct effects of cytokines like IFNα and IFNγ on hematopoietic cells (47, 48), several lines of experimental evidence support a role of the microenvironment in these processes.
  In the steady state, most hematopoietic stem cells are quiescent. Loss of quiescence and proliferation causes cumulative damage to HSC leading to their functional exhaustion (38, 39, 47, 48). Kobayashi et al., demonstrated that treatment with the bacterial second messenger c-di-GMP induced emergency myelopoiesis and loss of HSC quiescence. In agreement, c-di-GMP also caused a three-fold loss of bone marrow HSC without impairing surviving HSC function. Mouse chimera experiments indicated that loss of HSC required expression of STING—the c-di-GMP receptor—in both hematopoietic and non-hematopoietic cells (49).
  Many infections cause loss of B cell lymphopoiesis (50–53). B cell production in the bone marrow is maintained by CXCL12 and IL7-producing perivascular cells and CXCL12 producing osteoblasts (4, 6, 12, 54). Injection of adjuvants mimic the suppression of lymphopoiesis observed during infection (50, 55). Using this experimental paradigm, Ueda et al., showed that TNFα caused reduction of CXCL12- which is produced only by stromal cells in the bone marrow- leading to B cell egress from the BM (55). Similarly, the Link Laboratory showed that G-CSF targets monocyte-lineage cells and this in turn induces downregulation of CXCL12, IL7, and other B cell supportive cytokines in perivascular stromal cells and osteoblasts (56, 57). Note that the same studies also observed that G-CSF treatment depleted perivascular cells and osteoblasts (56, 57). In agreement, Terashima  et al., found that sepsis-induced G-CSF release caused loss of osteoblasts, reduced production of IL7 by the surviving osteoblasts, and depletion of common lymphoid progenitors leading to inefficient lymphopoiesis (52). Together these studies suggest that infections that cause increases in TNFα and/or G-CSF in the bone marrow will suppress lymphopoiesis by directly destroying the niche and inhibiting the ability of the surviving niche cells to support lymphopoiesis. The bone marrow is also the main reservoir for long-lived plasma cell and memory T cells (58–60). Both cell types are in contact with CXCL12- and IL7-producing perivascular cells (54) and require CXCL12 and other microenvironment-produced signals for maintenance in the BM (61). These suggest that the G-CSF mediated destruction/inhibition of the microenvironment described above will also impact the ability of the BM to recruit and maintain plasma cells and memory T cells.
4. Infection-induced remodeling and damage of the microenvironment: The bone marrow microenvironment is remarkably dynamic and can be extensively remodeled after myeloablation (62, 63), aging (64), and leukemia (35, 65). Infection is no exception and several pieces of data indicate extensive injury and remodeling of bone marrow stromal populations in response to infection or stimulation with bacterial cell wall components.
  It is becoming clear that endothelial cells and blood vessels in the bone marrow undergo rapid angiogenesis and remodeling in response to infection. Scumpia et al., observed dilated sinusoids as soon as 12h after cecal ligation in a mouse model of sepsis, suggesting that infection affects bone marrow vessel permeability (66). Many infections lead to increased levels of IFNα. Prendergast et al., found that pIpC treatment (an IFNα inducer) or IFNα injection caused a threefold increase in the number of BM endothelial cells, upregulation of adhesion molecules, vascular dilation, and permeability in wild-type but not *Ifnar^−/−^* mice. Surprisingly, mouse chimera experiments showed that IFNAR expression in stromal cells or hematopoietic cells is sufficient to activate the endothelium (67). In agreement, Vandoorne et al., found increased sinusoids, endothelial proliferation, and angiogenesis, and increased vascular permeability in response to LPS (68). Increased permeability correlates with neutrophil egress from the BM (68), suggesting that vascular remodeling facilitates mobilization. Non-endothelial stromal cells are also remodeled by infection. As discussed in point three, G-CSF-mediates the destruction of CXCL12-producing perivascular cells and osteoblasts (52, 56, 57). Additionally, c-di-GMP—which targets stromal cells *via* STING to cause loss of HSC (49)—also caused vascular dilation and loss of endothelial cells and perivascular stromal cells and that this required STING expression in stromal cells. The specific mechanisms for this stromal destruction are not known, but c-di-GMP upregulates G-CSF (49), strongly suggesting a role for this cytokine in remodeling the microenvironment in this experimental paradigm. In addition to these *in vivo* studies, there is evidence showing that many pathogens can directly infect endothelial and BM stromal cells and reduce their ability to support hematopoiesis *in vitro* (69–73). Together these studies demonstrate that infection massively remodels the microenvironment that supports hematopoiesis—likely *via* direct and indirect mechanisms—and suggest that destruction of the microenvironment might be a major driver for loss of hematopoietic function during infection.

## Discussion

The manuscripts discussed above provide overwhelming evidence for the role of the microenvironment in orchestrating the bone marrow response to infection. However, a common limitation is that most studies have focused on one aspect of the response to infection (emergency myeloid cell production, mobilization, hematopoietic and stromal injury). Thus it is still not possible to know if these are aspects of a single response controlled by common pathways (e.g., G-CSF) or individually controlled processes that allow fine-tuning of the response through the kinetics of the infection. The mechanisms through which infection damages the stromal compartment and how these structures regenerate are highly interesting; especially in light of recent scRNAseq studies demonstrating that the stromal compartment of the bone marrow is highly heterogeneous (35, 74, 75); and suggesting that specific components of the microenvironment provide unique niches supporting the differentiation of distinct lineages (1, 4, 6, 10, 12, 75). Whether some infections preferentially affect some niches but not others remains open. It is well established that aging negatively impacts HSC function, biases hematopoiesis toward myeloid cell production, and dramatically remodels the microenvironment (64, 76). The answers to these questions will provide critical insights into how the bone marrow functions during stress, and lead to the development of new therapies to preserve/improve bone marrow function during infectious challenges.

## Author Contributions

CBJ, JZ, and DL conceived and wrote the manuscript. All authors contributed to the article and approved the submitted version.

## Funding

This work was partially supported by the National Heart Lung and Blood Institute (R01HL136529 to DL).

## Conflict of Interest

The authors declare that the research was conducted in the absence of any commercial or financial relationships that could be construed as a potential conflict of interest.
